# Gabapentin Effect on Pain Associated with Heroin Withdrawal in Iranian Crack: a Randomized Double-blind Clinical Trial

**Published:** 2012

**Authors:** Behnaz Behnam, Vahid Semnani, Nadia Saghafi, Raheb Ghorbani, Mina Dianak Shori, Samaneh Ghooshchian Choobmasjedi

**Affiliations:** a *Psychiatry Group, Semnan University of Medical Sciences, Semnan, Iran.*; b *Pathology Department, Semnan University of Medical Sciences, Semnan, Iran.*; c *Epidemiology Group, Semnan University of Medicine, Semnan, Iran.*; d *Semnan University of Medical Sciences, Semnan, Iran. *

**Keywords:** Gabapentin, Heroin, Iranian crack, Pain

## Abstract

Gabapentin seems to be a safe and well tolerated medication for treating heroine dependence. This study examined the efficacy of gabapentin for relieving withdrawal-related pain due to heroin use.

Sixty men were recruited from an inpatient psychiatric ward of Fatemieh hospital in Semnan and randomized to receive either placebo (n = 30) or gabapentin (1800 mg/day) (n = 30) for 7 days. Subjective Opioid Withdrawal Scale (SOWS) was measured as a self-administered scale for grading body pain at baseline, and on days 1, 2, 3, 4, 6, and 7.

Mean of pain score had a significant decrease trend in both gabapentin and placebo groups. Pain severity during the most of detoxification duration was significantly lower in gabapentin group compared with the placebo group.

It is suggested that gabapentin may have an effective role in removing heroin withdrawal-related pain.

## Introduction

Gabapentin is a gamma-Aminobutyric acid (GABA) analogue that was originally developed for the treatment of epilepsy, pain relief, and especially neuropathic pain (1). Results with human and rat brain Nuclear magnetic resonance (NMR) spectroscopy indicate that gabapentin increases GABA synthesis, probably by modulating, as it does *in-vitro*, the action of the GABA synthetic enzyme, glutamic acid decarboxylase and the glutamate synthesizing enzyme, branched-chain amino acid transaminase (2). Gabapentin is one of the most common medications used in addicted ones to alcohol and other substances. It has been suggested that gabapentin when taken for 40-60 days in doses of 1500 mg at bedtime, is effective in reducing Alcohol craving (3-5). It also attenuates the severity of withdrawal symptoms experienced by those physically dependent on opioid analgesics, such as heroin, morphine, and methadone (6). Some studies also demonstrated a significant reduction in the severity of cocaine withdrawal syndrome (7-9). 

Dependence to crack, the chemical cocaine hydrochloride, is one of society›s greatest universal problems in most countries, especially developing societies. It has penetrated all levels of each society and even no area of the developed countries is crack-free. Not only, the chances of overdosing and poisoning leading to coma, convulsions, and death are greatly increased, psychologically, the drug reduces concentration, ambition, drive, and increases confusion and irritability, wreaking havoc on users’ professional and personal lives (10). Although used crack in western countries potentially includes cocaine, but consumed crack in Iran (named Iranian crack) contains condensed heroin. In fact, its main material is originated from heroin.

Although multiple medications have been studied for the treatment of dependence to various substances, no medications have been shown to have a robust effect on Iranian crack craving and its-related side effects. This study examined the efficacy of gabapentin in patients undergoing inpatient treatment for Iranian crack (containing heroin) withdrawal-related pain.

## Experimental

Sixty men were recruited from an inpatient psychiatric ward of Fatemieh hospital in Semnan after reviewing medical records. Subjects 18 to 60 years old were eligible if they met DSM-IV TR criteria according to the American Psychiatric Association guideline for current substance dependence as well as based on psychiatrist diagnosis. All patients had the history of regular Iranian crack usage at least within the last year. Patients with major psychological disorders, acute physical disorders, or brain organic disease were excluded from the study. Blood tests to rule out electrolytes and metabolic abnormalities included sodium (Na), potassium (K), blood sugar (BS), thyroid stimulating hormone (TSH), renal function tests (BUN and Cr), and liver function tests (AST, ALT, AlkPh, Bil) were done. All electrolytes were measured using biochemistry kit (Zistchimi, Iran), thyroid parameters were measured using Diaplus Kit (Diaplus, USA), serum bilirubin levels were measured using Parsazmoon biochemical kits (Iran), and other biomarkers were measured using Man kit (Man, Iran).

The study protocol and recruitment procedures were approved by the Review Board of the Semnan University of Medical Sciences. Written informed consent for the full protocol was obtained.

All subjects qualified by taking similar doses of clonidine (Tolidaroo, Iran; 0.2 mg/q8h at the first day, 0.3mg/q8h at the second day, 0.4 mg/q8h at the other days), Amitriptyline (Daroopakhsh, Iran; 75 mg on the first day, 100 mg on the second day, 150 mg on the third day, 200 mg on the fourth day and 300 mg on the other days), Ibuprofen (Tehranshimi, Iran; 800 mg/q6h), Hydroxyzine (Poorsina, Iran; 100 mg/q6h), Chlorpromazine (Tehranshimi, Iran; 100 mg/q6h), Hyoscine (Tehranshimi, Iran; 20 mg/q6h), and Lorazepam (Abidi, Iran; 2 mg/q6h).

The subjects were systematically randomized (EpiInfo program, WHO and CDC, Version 6.4) to receive either placebo (n = 30) or gabapentin (n = 30) for 7 days. Study investigators, raters, and subjects were also blinded to treatment assignment until all study visits were completed and the data set was cleared. Subjects were assessed at baseline, and on days 1, 2, 3, 4, 6, and 7.

During the double-blind phase, study medication was titrated to thirty capsules of either gabapentin (Irandaroo, Iran) or placebo orally 45 min prior to bedtime over a 7-day period. Each capsule received by the active medication group contained 300 mg of gabapentin (one capsule) for the first day and then 300 mg/q12h for the second day, 300 mg/q8h for the third day, and 600 mg/q8h for the other days. Each subject received one capsule at bedtime for three nights, then two capsules at bedtime for four nights. On day 7, subjects were reassessed by the study physician.

Subjective Opioid Withdrawal Scale (SOWS) was measured as a self-administered scale for grading opioid withdrawal symptoms. This scale assesses the intensity of 16 symptoms which is rated by patients on a scale of 0 (not at all) to 4 (extremely). Among all components, we used the item of body pain for assessing the severity of pain in both the gabapentin and placebo groups (11).

Data are expressed as mean ± standard deviation (SD) for quantitative variables and were summarized by absolute frequencies and percentages for categorical variables. Continuous variables were compared using t-test or non-parametric Mann-Whitney U-test whenever the data did not appear to have normal distribution or when the assumption of equal variances was violated across the groups. Categorical variables across the two groups were compared using the chi-square test or Fisher›s exact test if required. We considered 2-tailed p*-*values of ≤ 0.05 as statistically significant. Analyses were performed using SPSS statistical software (version 16.0) for windows. 

## Results and Discussion

**Figure 1:**
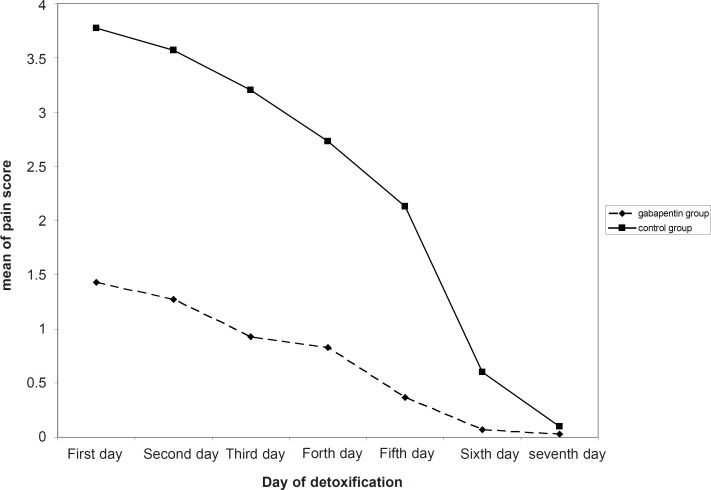
Trend of mean pain score in the two gabapentin and placebo group within the detoxification period

In current study, 30 men (aged 26.5 ± 5.1 years) were treated with gabapentin and another 30 (aged 27.6 ± 7.5 years) were administered placebo. No significant differences were observed on age, measures of used Iranian crack, as well as duration of it across the two groups (Table 1). As shown in Figure 1, mean of pain score had a significant decreasing trend in both gabapentin and placebo groups. Pain severity during the first five days of detoxification was significantly lower in gabapentin group compared with the controls (Table 2). 

**Table 1:** Baseline characteristics in gabapentin and placebo group

**Characteristics **	**Gabapentin group (n = 30) **	**Placebo group (n = 30) **	**p-value **
Age (year)	26.5 ± 5.1	27.6 ± 7.5	0.522
Measure of used crack (g/day)	0.87 ± 0.39	0.78 ± 0.24	0.327
Duration of crack using (year)	2.8 ± 1.1	3.0 ± 0.9	0.522

**Table 2:** Severity of pain based on SOWS scale in gabapentin and placebo group

**Characteristics**	**Gabapentin group (n = 30)**	**Placebo group (n = 30)**	**p-value**
First day	1.43 ± 1.04	3.77 ± 0.50	< 0.001
Second day	1.27 ± 0.74	3.57 ± 0.57	< 0.001
Third day	0.93 ± 0.74	3.20 ± 0.76	< 0.001
Forth day	0.83 ± 0.70	2.73 ± 0.69	< 0.001
Fifth day	0.37 ± 0.62	2.13 ± 0.86	< 0.001
Sixth day	0.07 ± 0.37	0.60 ± 0.93	0.003
Seventh day	0.03 ± 0.18	0.10 ± 0.31	0.305

Several controlled clinical trials in various diseases subgroups showed that gabapentin at 2400-3600 mg/day has an efficacy for inhibiting neuropathic pain including cancer-related pain, pain associated with HIV infection, chronic back pain and others (12, 13). Gabapentin as a GABA analogue can also provide new avenues for pharmacological treatment of substances dependence. This drug is an antiepileptic shown to be effective in the treatment of pain disorders and appears to be useful for several psychiatric disorders as well as alcohol withdrawal and cocaine dependence. It has been indicated that gabapentin, at a dose of 600 mg three times a day, appear to lead an effective pain relief and an overall beneficial effect on symptoms of drugs withdrawal. Among these substances, exposure to crack can result in experiencing painful and life threatening withdrawal, irritability, poor ability to regulate body temperature and increased risk of having seizures. Therefore, it seems that administration of gabapentin with appropriate dosages can effectively inhibit adverse events of drug misuse (14). The present study suggests an effective role for gabapentin in removing Iranian crack, heroin, withdrawal- related pain. As can be seen in Table 2, this effect is statistically significant until day 6; in days 6 and 7 the mean of pain score is markedly decreased in both gabapentin and control groups and is almost disappeared, so the difference in pain scores is decreased in the 6^th^ day and not seen in the 7^th ^day. Similarly, Myrick *et al. *reported a reduced amount and frequency of the use of cocaine following administration of gabapentin (15). Also, in a study by Foltin *et al*., the highest dose of gabapentin tested (1200 mg/day) decreased the discriminative stimulus effects of cocaine and decreased cocaine craving by 41–53% following cocaine administration (16). Resent findings support that gabapentin as prescribed for the treatment of neuropathic pain, is effective in decreasing opioid-induced pain hyperalgesia (17). Furthermore, there are some evidences showing that gabapentin is effective in neuropathic pain, whereas other authors could not demonstrate the role of gabapentin for pain relief. Bisaga *et al. *in a study on individuals with cocaine dependence observed that gabapentin 1600 mg bid was no more effective than placebo in the treatment of cocaine dependence (18). Available evidences mainly focused on the gabapentin effectiveness for relieving acute pain (19) and its beneficial influence on chronic pain conditions was not proved in most of them.

Xin Wei showed that gabapentin can significantly prevented opioid-induced hyperalgesia (OIH) induced caused by fentanyl and morphine, suggesting a role for the addition of gabapentin in the perioperative period and during chronic pain treatment as an effective symptoms of heroin withdrawal drug to prevent OIH (20). Individuals on methadone maintenance for the treatment of addiction (MM) are demonstrated to be hyperalgesic to cold-presser pain in comparison to matched controls and ex-opioid addicts, a finding described as clinical evidence of opioid-induced hyperalgesia (OIH). Peggy Compton *et al. *showed the efficacy of a key pharmacotherapy for neuropathic pain, gabapentin (GPN), to reverse OIH in MM patients (21). Following detoxification, patients experience severe back pain and restlessness often accompanied by a restless-leg-syndrome. Freye E *et al. *evaluated gabapentin given immediately following detoxification to attenuate these symptoms (22).

## Conclusion

In our survey, the effect of gabapentin to reduce withdrawal-related pain due to Iranian crack heroin use was confirmed. Hence, use of this drug for management of such condition is recommended. 
